# Adsorption of Copper and Arsenic from Water Using a Semi-Interpenetrating Polymer Network Based on Alginate and Chitosan

**DOI:** 10.3390/polym15092192

**Published:** 2023-05-05

**Authors:** Mohammad T. ALSamman, Julio Sánchez

**Affiliations:** Departamento de Ciencias del Ambiente, Facultad de Química y Biología, Universidad de Santiago de Chile (USACH), Santiago 9170022, Chile

**Keywords:** adsorption, alginate, arsenic, chitosan, copper, water treatment

## Abstract

New biobased hydrogels were prepared via a semi-interpenetrating polymer network (semi-IPN) using polyacrylamide/chitosan (PAAM/chitosan) hydrogel for the adsorption of As(V) or poly acrylic acid/alginate (PAA/alginate) hydrogel for the adsorption of Cu(II). Both systems were crosslinked using N,N′-methylenebisacrylamide as the crosslinker and ammonium persulfate as the initiating agent. The hydrogels were characterized by SEM, Z-potential, and FTIR. Their performance was studied under different variables, such as the biopolymer effect, adsorbent dose, pH, contact time, and concentration of metal ions. The characterization of hydrogels revealed the morphology of the material, with and without biopolymers. In both cases, the added biopolymer provided porosity and cavities’ formation, which improved the removal capacity. The Z-potential informed the surface charge of hydrogels, and the addition of biopolymers modified it, which explains the further metal removal ability. The FTIR spectra showed the functional groups of the hydrogels, confirming its chemical structure. In addition, the adsorption results showed that PAAM/chitosan can efficiently remove arsenic, reaching a capacity of 17.8 mg/g at pH 5.0, and it can also be regenerated by HNO_3_ for six cycles. On the other hand, copper-ion absorption was studied on PAA/alginate, which can remove with an adsorption capacity of 63.59 mg/g at pH 4.0, and the results indicate that it can also be regenerated by HNO_3_ for five cycles.

## 1. Introduction

### Impact of Metal Ions on Human Health

Arsenic is a toxic element that is harmful to humans and a major pollutant in drinking water. The World Health Organization (WHO) and the US Environmental Protection Agency (EPA) set the guideline value for the maximum level of arsenic contamination in drinking water as 10 μg/L [1]. Chemically, there are different types of arsenic, and As(V) and As(III) are commonly found in groundwater in many countries, such as India, Bangladesh, Vietnam, and Chile. These contaminants are of great concern in developing countries as they can be released into water bodies from both natural and anthropogenic sources. Arsenic contamination can be geological or stem from human sources, including mining activities, the use of pesticides and fertilizers, and coal combustion. Arsenic is a complicated metalloid, and its adsorption is a very costly and difficult process. A common practice among researchers is to improve the adsorption capacity and increase the rate of adsorption for advanced treatment of water and wastewater [2,3].

Copper is a transition metal and participates in the process of electron chelation with appropriate ligands; for example, the COOH functional groups of the biopolymer (alginate) form complexes with metal ions (Cu^2+^) and increase the adsorption on the surface by forming a covalent bond [4,5]. Copper ions can irritate the nose, mouth, and eyes and cause headaches, stomach aches, dizziness, vomiting, and diarrhea. A large intake of copper can cause liver and kidney damage, and even death. Copper has a high density relative to water. Copper is also classified as a heavy metal, which includes many other metals and nonmetals, such as mercury, cadmium, arsenic, chromium, and lead [6,7,8].

**Figure 1:**
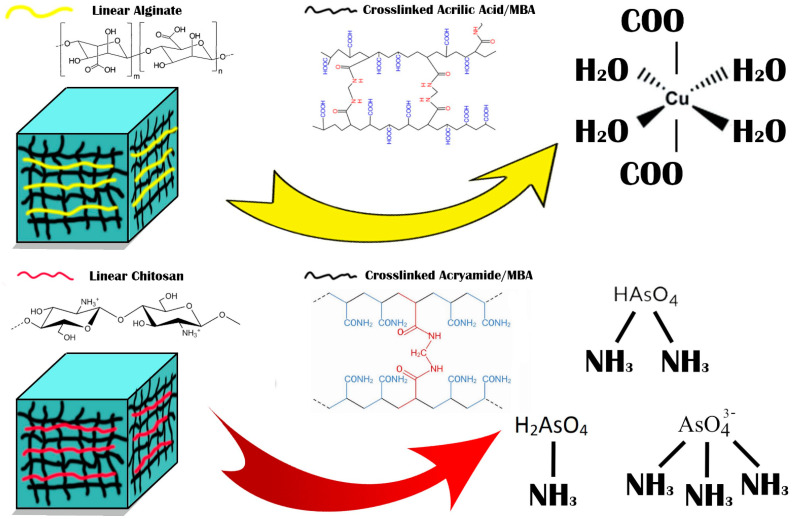
Hydrogels of PAA/alginate and PAAM/chitosan for adsorption of copper and arsenic.

Biopolymers can be combined with synthetic polymers to prepare novel materials with improved features toward metal ion removal. The best biopolymers are alginate and chitosan, and studies have shown that they are important in removing heavy metals, semimetals, and other ions from wastewater (see Figure 1).

Chitosan is a highly abundant deacetylated product of chitin, second only to cellulose, of the polysaccharides’ family, which are known to be low-cost, environmentally friendly, and sustainable biopolymers. Chitosan is composed of 1,4 glycoside bonds of 2-amino-2-deoxy-β-d-glucopyranose units, and is structurally a copolymer consisting of β-(1 → 4)-2-acetamido-d-glucose and β-(1 → 4)-2-amino-d-glucose, containing three types of reactive functional groups: an amino group (-NH_2_), amide (-CH_2_CONH^−^), as well as primary and secondary hydroxyl groups (-OH), at positions C2, C3, and C6 [9].

Chitosan has been used in sustainable biosorption to remove arsenic from polluted water due to the hydrophilic functional groups, -OH and -NH_2_, on its structure that are active sites for arsenic adsorption and where the amino groups of chitosan become protonated (i.e., become -NH_3_^+^). The electrostatic repulsion and electrostatic interactions in the process of arsenic adsorption occur via electrostatic affinity between the amino group (NH_2_) and the primary and secondary hydroxyl groups (CH_2_OH, CHOH) of chitosan and arsenic [10,11,12,13,14]. Chitosan is entirely produced by de-acetylating chitin in an alkaline media, is a linear biopolymer that can be derived from marine or animal sources, and is a semi-crystalline polysaccharide that contains N-acetyl-d-glucosamine and d-glucosamine residues organized in a linear dimension and can be utilized as a membrane, fibers, hydrogels, and sponges for water filtration [15,16]. The conventional source of chitosan is leftover crustacean shells from the seafood processing industry. It has a chemical structure that is comparatively stable and has the attributes of being polycationic, harmless, non-toxic, and biodegradable [17]. Due to the amino group (-NH_2_) in its structure, it has a cationic character, and this positive charge aids in the formation of an extracellular matrix to attract by drawing negatively charged molecules, and it is most abundant, being the second-most prevalent polymer after cellulose [15,18].

Modified chitosan films were prepared to remove arsenic at an initial pH < 4 and a contact time of 24 h. The initial concentration varied from 5 to 500 mg/L, with a removal capacity of 10.4 mg/g, which is more than six times higher than the chitosan film, and the absorption when using the chitosan film for solutions reached 1.6 mg/g [19].

Another example is the use of nanoabsorbent iron oxide/N-isopropyl acrylamide/chitosan, which was manufactured through a polymer grafting process, and then was used for the removal of arsenic using 10 mg of gel and a concentration of 10 μg/mL arsenic at pH 7.5, a contact time of 60 min, and a temperature of 45 °C. The removal capacity was 2.7 mg/g, and the removal efficacy was 82.2% [20].

Alginate is a polysaccharide biopolymer extracted from brown seaweed consisting of anionic blocks of (1 → 4) linked to glucuronic acid (G) and mannuronic acid (M). Both structural units contain carboxyl groups (-COO) and many hydroxyl groups (-OH) on the molecular chain. Alginate (alginate salts) shows a high affinity for metal ions, and excellent biodegradability, biocompatibility, and environmental friendliness [21]. The efficiency of Cu(II) removal is related to the -COOH groups present in acrylic acid (AA) and the carboxyl groups (-COO) of alginate, but due to its tendency to swell in water and other mechanical weaknesses, its absorbance remains limited [22,23,24]. The literature shows examples of gels fabricated from a mixture of acrylic acid and chitosan using the irradiation method by preparing 20% acrylic acid with 0.5% *w/v* chitosan in a vessel on a magnetic stirrer for 1 h, and then irradiating the crosslinking with a dose of radiation. The resulting gels were cut into small pieces and soaked in KOH for one hour to increase the adsorption capacity. The adsorption capacity was 171 mg/g for Cu(II) at pH 5.5 for 24 h, with a removal efficiency of 99% [25].

Another example is the hybrid alginate oxide and graphene oxide gel beads. The following gel beads were formed: e(Ca-Alg2) and Ca-Alg2, with sorbent materials of coated graphene oxide (Ca-Alg2/GO) to absorb Cu(II) ions from aqueous solution with a pH of 5.5 in mM-Q water. The copper ion absorption capacities of Ca-Alg2 and Ca-Alg2/GO were found to be 42.7 mg/g and 60.2 mg/g gel granules, respectively. At the lowest concentration of 0.29 g/L, the removal efficiency was 13%, while at the highest concentration of 3.2 g/L, the removal efficiency was 77%. The highest concentration of Ca-Alg2/GO at 2.8 g/L showed a removal efficiency of 81% [26]. An example was reported concerning the ability of gels composed of chitosan nanoparticles and alginate microparticles to absorb Cu(II) from copper sulfate solutions at a constant pH of 3.5 at a concentration of 2 mg/mL. The absorption capacity was obtained as 112.4 mg/g [27]. In another study, gels of TiO_2_/ZnO hybrid calcium alginate beads were used to remove Cu(II) via hybrid photocatalysts using TiO_2_/ZnO to form TiO_2_/ZnO-CaAlg. Three different mass ratios of TiO_2_:ZnO were used for Cu(II) at pH 7. The results showed that the copper removal efficiency reached 98.9% using a solution of Cu(II) concentration = 20 mg/L [28].

According to the previously cited examples, the following scientific paper is related to the preparation and characterization of PAA/alginate hydrogels to absorb copper and PAAM/chitosan hydrogels to absorb arsenic. These biobased materials could reach a higher removal capacity of these metal(oid)s, such as arsenate and copper, compared to synthetic polymers. The use of biopolymers in the preparation of active hydrogels leads to opportunities for these novel biobased materials.

## 2. Materials, Methods, and Characterization

### 2.1. Reagents

Chitosan (85% deacetylation, CAS: 9012.76.4, Sigma-Aldrich, product of Iceland), sodium alginate (90% carboxylation, CAS: 9005.38.3, Sigma-Aldrich, product of China), acrylamide (CAS: 76.06.1, Sigma-Aldrich, made in China), acrylic acid (CAS: 79.10.7, Merck, made in Germany), N,N-methylenebisacrylamide (CAS: 110.26.9, Sigma-Aldrich, USA), ammonium persulfate (CAS: 7727.54.0, Sigma-Aldrich, product of Turkey), copper standard solution (product 1.19786, Merck KGaA, made in Germany), arsenic standard solution (product 1.19773, Merck KGaA, made in Germany), nitric acid 65% (CAS: 7697-37-2, Merck KGaA, made in Germany), hydrochloric acid 37% (CAS: 7647-01-0, Merck KGaA, made in Germany), and sulfuric acid (10 N) (CAS: 7664-93-9, made in Germany) were used.

### 2.2. Adsorbent Synthesis

Initially, acrylamide was weighed, and then different percentages of chitosan, MBA, and PSA were added to Schlenk tubes. MBA and PSA were added to another Schlenk tube using the same process, but with acrylic acid and different percentages of alginate (see Table 1). Then, the resulting gels were kept in a refrigerator and placed in dryers. The gels were ground, and a usable powder was obtained.

SEM samples were prepared by taking these samples and drying them through a lyophilizer, after which the samples are plated with gold so that they did not burn due to scanning electrons on the surface. The scanning process was conducted, and then pictures of the samples were taken at a magnification capacity of X100, X200, X500, and X1000, with a length of 10–100 μm.

In the case of Z-potential analysis, the charges can be studied by placing the samples in water and sodium nitrate salt, with a pH of 4–7, and then measuring and testing them. The samples were studied at low and high pH values.

Concerning metal ions, the two biobased hydrogels were applied in the removal experiments, and the following variables were considered: appropriate percentage of biopolymer, quantity of adsorbent, optimum pH, contact time, concentration of metal ions, reuse study, and effect of elution. The removal efficiency was calculated according to Equation (1). Additionally, the adsorption capacity of metals was calculated according to Equation (2), where *C*0 is the solution at the initial time and *Ct* is the concentration at equilibrium (mg/L), *m* (g) is the mass of the adsorbent, and *V* (mL) is the volume of the solution. The concentration of metal ions was determined by atomic absorption spectroscopy (Perkin Elmer) [29].

(1) $$Removal\;effeiciency\left(\%\right)=\frac{C0-Ct}{C0}\times 100$$

(2) $$qt\left(\frac{mg}{g}\right)=\frac{\left(C0-Ct\right)\times V}{m}$$

## 3. Results and Discussion

### 3.1. Scanning Electron Microscopy (SEM)

**Figure 2:**
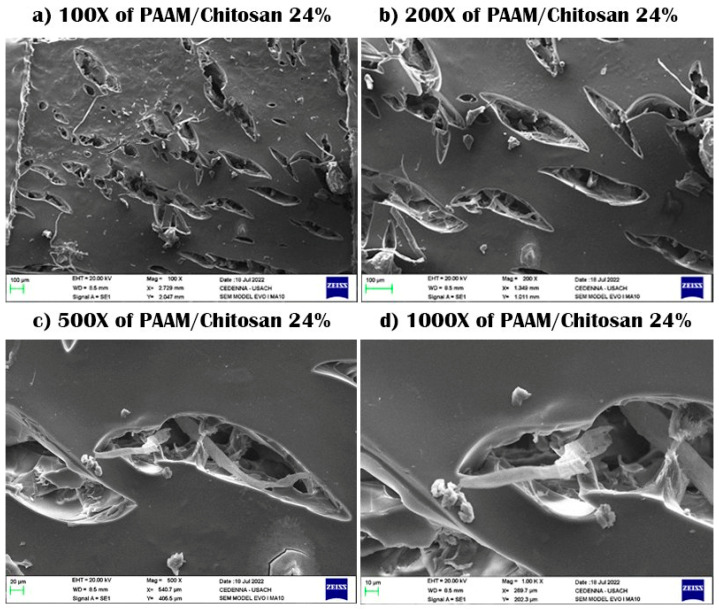
SEM images of PAAM/chitosan 24% xerogels using 100× to 1000× magnification.

**Figure 3:**
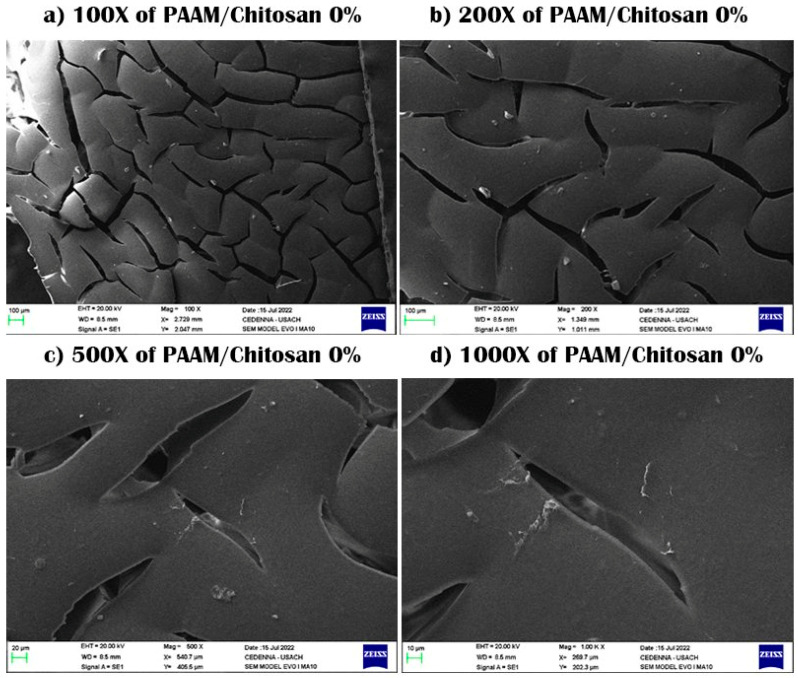
SEM images of PAAM/chitosan 0% xerogels using 100× to 1000× magnification.

SEM was used to investigate the morphology, surface, and formation of polysaccharide-based hydrogels. The cross-sectional surface showed a very rough surface, with characteristic jagged edges, large wrinkles, and fissures due to the partial breakdown of the polymeric gel network during the drying process, causing cracks and wrinkles for both PAAM/chitosan 0% and 24% [30]. We note that the presence of chitosan could be observed as a linear segment on the surface, and pores and gaps were also observed in these two semi-IPN gels. Chitosan contains hydroxyl (OH) and amine (NH_2_) polar functional groups in its structure, and it forms a linear polymer that does not chemically contact acrylamide. The surfaces of the hydrogel appeared rough and were interspersed with many cracks, and we noticed that they became rougher on acrylamide gels without chitosan. SEM images of PAAM/chitosan samples were obtained at magnifications of 100× and 1000× (see Figure 2a–d) [31,32]. It appears that the physical aspect of chitosan particles remained unchanged. However, the SEM images at 1000× magnification showed that the surface texture of acrylamide was significantly different from that of chitosan, indicating that it was not chemically modified and that it was not crosslinked, which proved the linear structure of chitosan (see Figure 1). We also noticed some cracks, but they were due to the shearing process during processing of the sample or inflation-specific surface areas for chitosan and acrylamide [33,34]. Figure 3 shows the SEM images of PAAM/chitosan 0%.

**Figure 4:**
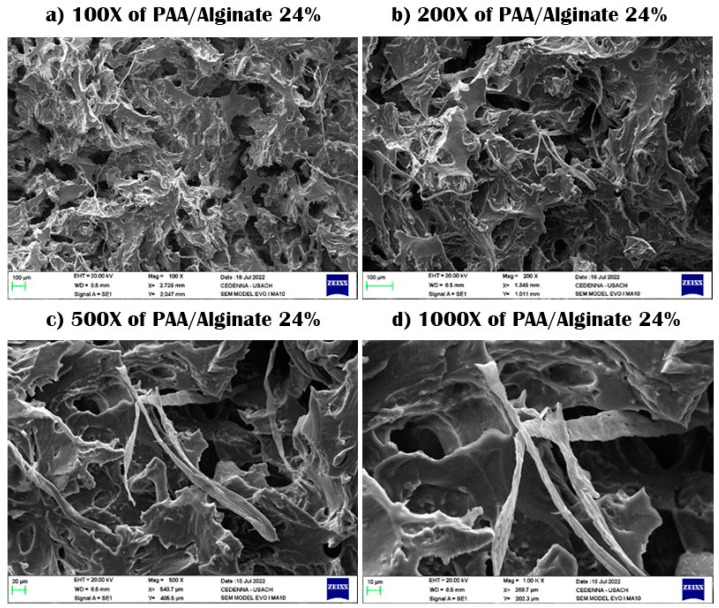
SEM images of PAA/alginate 24% xerogels using 100× to 1000× magnification.

In the case of the PAA/alginate system, the images showed alginate aggregates, which were linear polymers, and this pore pattern was observed in the rest of the hydrogel groups observed in semi-IPN, as shown in Figure 4. It can also be seen that there was no clear phase separation, which indicates the homogeneous distribution of components in composite hydrogels, and the micrographs showed a rougher morphology, with an increased percentage of alginate as the surface roughness. It also increased the generation of pores and gaps in the gels and had a significant influence on the surface morphology of the superabsorbent material [35,36,37,38].

As an example similar to our case, in one study it was found that the poly(acrylic acid)-g-sodium alginate hydrogel has a three-dimensional porous structure. These pores may be generated by the electrostatic repulsion between the alginate carboxyl anion (-COO^−^) during the polymerization process and showed morphology with high-density crystalline growth on the surface with different pore sizes [39].

In another study, the structure of the hydrogels was studied through SEM analysis to study the surface morphology of poly(acrylic acid)-grafted sodium alginate SA-cl-PAA and poly(acrylic acid) (PAA) grafted on sodium alginate SA-clPAA. The surface morphology in the case of the SA hydrogel was smooth, compact, and flat, with a crystalline and hard surface, and SA-cl-PAA and SA-clPAA were formed by adding AA plugs to SA. A rough, porous, and loose surface was formed, resulting in a hydrogel system containing an expanded molecular cloud shape, with the difference between the two hydrogels being SA-cl-PAA. SA-clPAA only affected the roughness [40].

In yet another study, sodium alginate-grafted poly(acrylic acid) hydrogels (SA-g-pAA hydrogels) with glycol dimethacrylate (EGDMA) as a crosslinker hydrogel sample were analyzed by SEM to study the surface morphology and surface features. The surface studies showed that the surfaces were intact for hydrogels with different magnifications, and they featured a smooth and solid outer surface with no porosity on it and a dense structure due to the presence of weak attractive forces between the polymer and the monomer, which led to a good compatibility between the contributors (SA and AA) [40].

**Figure 5:**
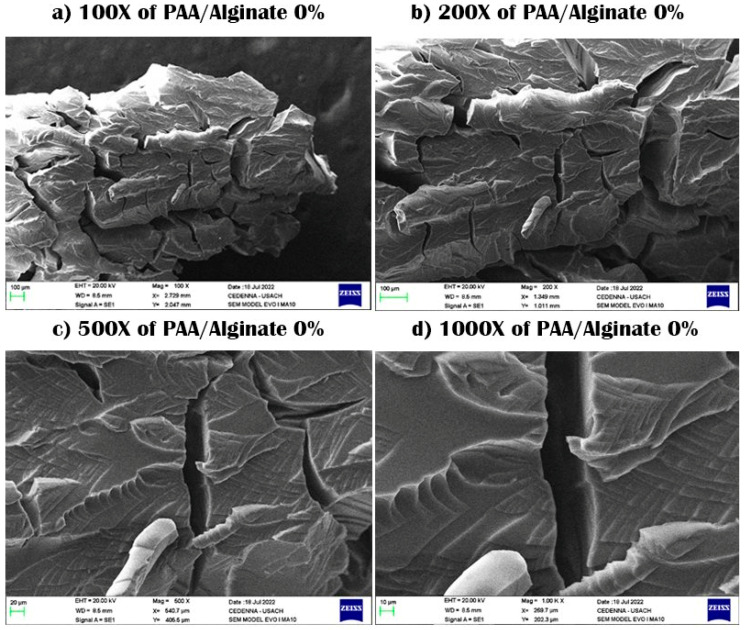
SEM images of PAA/alginate 0% xerogels using 100× to 1000× magnification.

After examining the SEM data, small folds could be observed on the 1000× SEM micrographs throughout the entire PAA matrix (see Figure 4a–d). The distance between particles was approximately 20–50 µm [35,41]. The images reveal the formation of a macroscopic network on the surface of the sample with a pore diameter of 10 to 20 µm. Based on Figure 5, it can be seen that there was a smooth structure, with no linear dendrites of alginate, and a smooth structure without linear polymers [42,43]. The pore sizes remained within the micrometer range, and the pore structure was lower in the PAA network. The porous structure increased the surface area of the hydrogel when the gel was dipped into an aqueous medium, and the medium easily diffused into the gel through microporous gaps [44,45,46].

### 3.2. Zeta Potential Characterization

**Figure 6:**
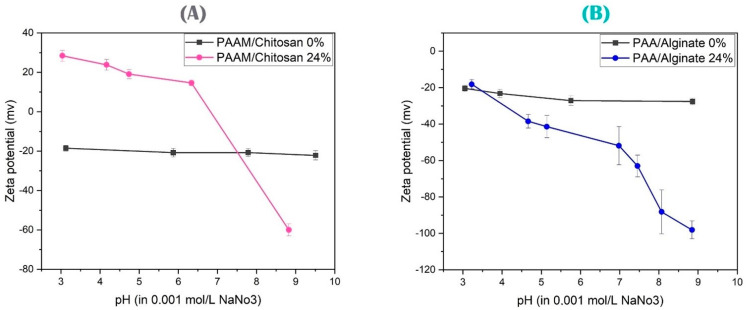
(**A**) Zeta potential value of PAAM/chitosan 24% and PAAM/chitosan 0% at different pH values. (**B**) Zeta potential value of PAA/alginate 24% and PAA/Alginate 0% at different pH values.

The Zeta potentials reveal the dependent charges of the gel compound. This study reveals the Zeta potentials of different types of chitosan- and alginate-based gels. Figure 6 illustrates the mechanism, which is a self-retarding action brought on by the anionic carboxyl of the adjacent group. The mechanism can be summarized as follows: A Schiff base must first be generated in order to produce acrylamide, showing that the hydrophobicity, nitrogen-to-carbon (N/C) ratio, and functional groups used to remove As(V) ions from the aqueous solutions using acrylamide were primarily controlled by the film diffusion mechanism, with a significant contribution from the intra-particle diffusion mechanism. These results suggested that the C=O, C-O, and N-H groups were the primary functional groups involved in the adsorption of PAAM/chitosan [47]. 

Considering poly(acrylic acid), the adsorption capacity of the composite can be influenced by the species of copper salts, which were in the following order: CuSO_4_ > CuCl_2_ > Cu(NO_3_)_2_ ≫ Cu(CH_3_COO)_2_. The chelation of COO^−^ with Cu(II) and the ion-exchange of COO-H^+^ with Cu(II), respectively, were attributed to the formation of the hydrogel [48].

As a potential adsorbent for extracting heavy metal ions from real wastewater, the PAA/alginate hydrogel has outstanding reusability and great operation performance. These polymers’ interaction mechanisms and common industrial applications are outlined. Many new uses for these polymers are possible in a variety of industries, including the production of paper and the treatment of water and wastewater. The stability or instability of the dispersion may depend on how the cationic polymers interact with other system elements, such as the inorganic/organic particles in aqueous dispersions. The three main mechanisms that can destabilize the particles are polymer bridging, charge neutralization, and polymer adsorption. The data obtained from the experiments on the adsorption process revealed that pH had a greater influence on efficiency than the other parameters. The created hydrogels contained the C-O, C double-bond O, and C double-bond C groups. The process of elimination was exothermic and spontaneous in terms of thermodynamics. Moreover, the thermodynamic studies demonstrated that the adsorption process progressed more favorably. The findings showed that the PAA/alginate might be considered as a practical and efficient sorbent for the removal of MB dye and Cu(II) from wastewaters. The ion-exchange process is responsible for the adsorption of PAA/alginate -COO^−^, and the main goal of this process is to create a coordination bond with heavy metals. The coordination chemistry theory has been used to explain the variations in the adsorption capacity of -COO. Moreover, heavy metals > H_2_O >> H^+^ demonstrate the adsorption selectivity of an expanding adsorbent containing COO^−^ [49,50,51]. According to our research, adding more crosslinkers to a typical absorbent resin can result in a material that is less expansive when it comes to adsorbing heavy metal contaminants. Upon adsorption, the hydrogen bond length was shorter, the population value was higher, and the delocalization of the gels on the same surface was stronger. The adsorption process on the surface was dominated by electrostatic interaction, whereas the adsorption mechanism on the surface was dominated by hydrogen bonding. The adsorption mechanism was found to be exothermic and spontaneous, and the hydrogel polymer maintained its initial adsorption capability during three repetitions of adsorption–desorption. In conclusion, the results demonstrate the possibility of cleaning up industrial effluent that has been tainted with metal ions [52,53,54,55,56,57,58].

In other words, the increased degree of hydrolysis significantly slowed down the rate of hydrolysis. This is listed as one of the causes of the polyacrylamide gel’s instability in the literature. The polymer needs to be stabilized, and the polymer’s solubility in the aqueous fraction decreased with increased hydrolysis. The polymer became more sensitive to the presence of electrolytes in the formation water at low levels of hydrolysis. A nucleophile addition took place, then the amine group was deleted and re-formed, and carboxylic groups were formed. When an acidic medium was added, the nucleophile attack of the amide group of hydrogen took place, and then a carboxylic group was formed as well. In this regard, electrostatic repulsion caused the pre-formed anionic carboxyl groups (-COO^−^) in the polymeric chain to exclude the electronegative nucleophilic groups (OH) from the chain. We note that acrylamide gels with chitosan had a positive charge, from the presence of protonated amine groups from chitosan, while acrylamide without chitosan had a negative charge. Chitosan has functional groups of amine, which will be protonated from NH_2_ to NH_3_^+^, demonstrating a positive charge, capable of absorbing anionic metals. At basic pH levels, the chitosan will face its deprotonation, demonstrating a negative charge. These results of +10 to +30 Zeta potential (mV) showed an incipient instability of the particles, but in the case of acrylamide, the amide groups were transformed into carboxylic groups -COOH, which turned into -COO^−^ (see Figure 6A) [59,60,61,62]. 

On the other hand, we note in Figure 6B that at acidic pH values, the results showed Zeta potentials of −30 to −40 (mV), which reflect the moderate stability of the particles at pH 4. We note that the carboxylic groups are -COOH in equilibrium with -COO^−^ [59,60], but in the case of alginates, we noticed a greater decrease in the Zeta potential, with a greater negative value, due to the abundance of carboxylic groups whose percentage is higher than PAA, where the Zeta potential of more than ±61 (mV) proved the excellent stability [61,62].

### 3.3. FTIR Characterization

**Figure 7:**
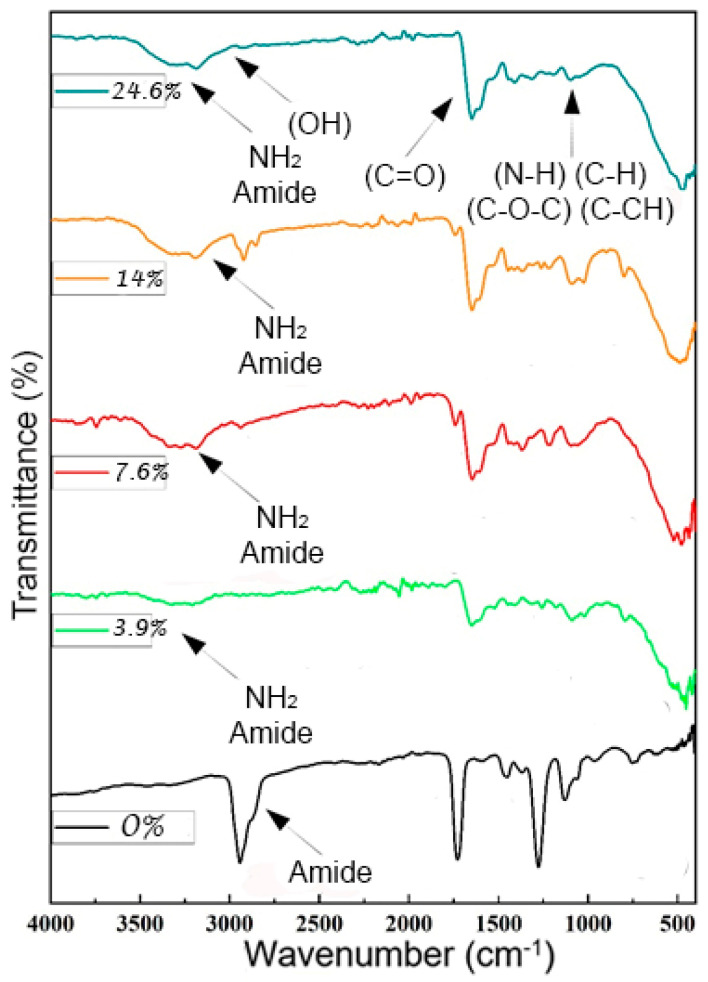
FTIR spectra of PAAM/chitosan xerogels containing different amounts of chitosan.

The crosslinked poly(acrylic amide) vibrations were visible in the spectra of PAAM/chitosan (0% to 24%) (see Figure 7), with the peaks appearing at 2947 cm*^−^*^1^ (amide 1) and 1732 cm*^−^*^1^ due to C=O stretching vibrations, and in the range between 1000 and 1500 cm*^−^*^1^. These bands (1139, 1232, and 1457 cm*^−^*^1^) are vibrations of C-H, C-CH, and N-H bending. Chitosan was added, which resulted in the formation of crosslinked chitosan. Amido bonds were created, and the amino bonds vanished, and they showed up in the spectra. The amino bonds vanished at a wavelength of 2952 cm*^−^*^1^; when bonds were established, an amide was created, and vibration bands owing to the saccharide structure (caused by O-H bending, C-O stretching, and C-N stretching) occurred at 956 to 1756 cm*^−^*^1^.

The inter-hydrogen bonds of the absorption peaks at 1500 and 1700 cm*^−^*^1^ corresponding to its carboxylic C=O stretching at 1657 cm*^−^*^1^ were responsible for the band that appeared in the wavenumber field of 2800 to 3500 cm*^−^*^1^ and could be assigned to the stretching vibration of O-H and the extension vibration of N-H [63,64]. Additional bands of N-H stretching were visible in the FTIR spectra at 1225 cm*^−^*^1^ (amide 1). At 2800 to 3800 cm*^−^*^1^, the combined O-H and N-H band of amide and amine stretching could be detected, and the band at 3310 cm*^−^*^1^ was caused by its hydroxyl (O-H) group [65,66].

**Figure 8:**
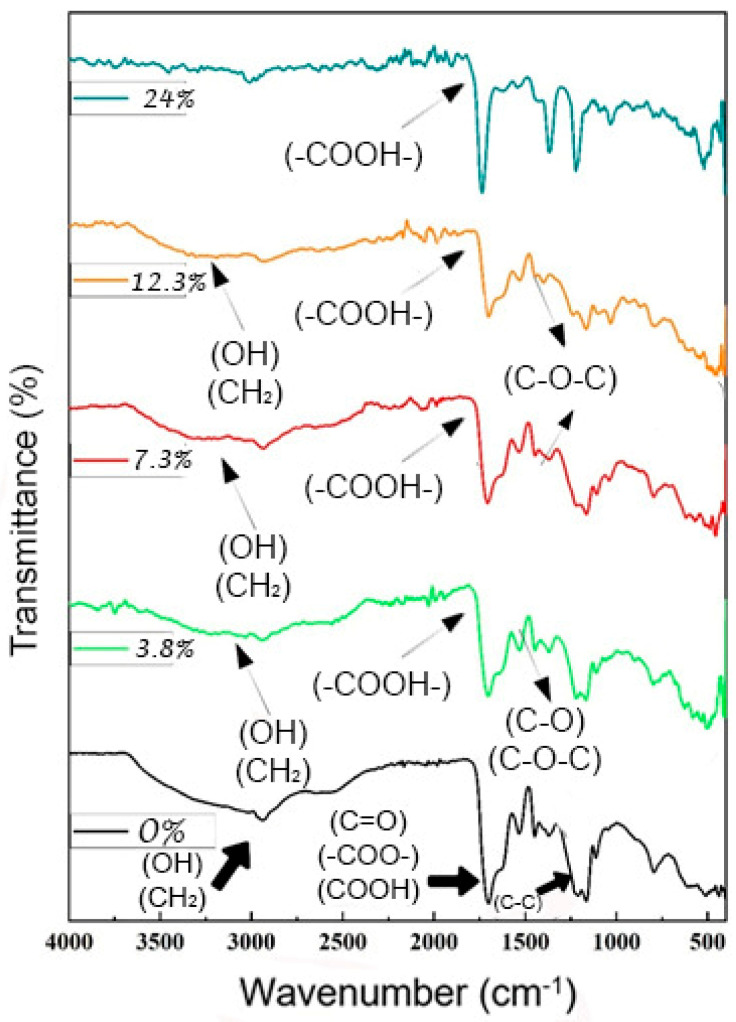
FTIR spectra of PAA/alginate xerogels containing different amounts of alginate.

In order to examine the PAA/alginate FTIR spectra shown in Figure 8, the differences between the spectra produced from samples with various quantities of alginate were compared.

The peaks for crosslinked PAA were in the absorption range of 1350 to 1850 cm*^−^*^1^ when sodium alginate was absent and the hydrogel sample was exclusively made up of crosslinked PAA [67]. The shoulders at 1710 and 1765 cm*^−^*^1^ were caused by the vibration of the carboxylate expansion C=O, and the range was 1700 cm*^−^*^1^. The COH bending of PAA caused the bands with peak values at 1541, 1454, and 1378 cm*^−^*^1^ to vibrate. The C=O expansion vibration of carboxylic PAA, which is proportional to the PAA concentration, was responsible for the range at 1700 cm*^−^*^1^, with shoulders at 1639 and 1705 cm*^−^*^1^, and hydrogen bonds are what created the bulging in the peaks [68]. We observed that there were no absorption bands at 1706 cm*^−^*^1^ (asymmetric COOH) and 1235 cm*^−^*^1^ (symmetric COOH), shifting at frequencies lower than 1750 and 1277 cm*^−^*^1^ when the biopolymer concentrations were altered [69]. In addition, we noticed that the C-O-C group of the anhydride seemed to be well-absorbed. Other domains that were impacted by anhydride creation can be attributed to C-O or O-H vibrations, which are the causes of the oscillations brought on by hydrogen bonding and are occasionally related to C-H oscillations. Another significant peak at 1172 cm*^−^*^1^ that was connected to the symmetric stretching mode of the carboxylate anion confirmed that the extremely strong characteristic band at 1706 cm*^−^*^1^ is attributable to stretching in the carboxylate anion. Additionally, the hydrogel’s FTIR spectrum and the partial hydrogel’s spectra were compared. The peaks shown in the figure were similar, but when sodium alginate was present in large quantities and was highly concentrated, three peaks that express the functional group’s formula appeared, with absorption ranges of 1141 and 1268 cm*^−^*^1^ due to the extended vibration of the C-OH group and 1644 cm*^−^*^1^ due to the vibration of its carboxylic group (COOH), which has an asymmetric expansion. As the hydrogel builds up a layer, this layer covers and weakens the hydrogen bonds, making the hydrogel extremely pliable [70]. As the amount of the biopolymer increased, the peak disappeared from 2310 to 3700, which is proof of the development of semi-IPN, the presence of etheric bonds, and the disappearance of OH groups.

### 3.4. Removal of Arsenic and Copper

#### 3.4.1. Effect of Biopolymer Amount on Metal Removal

The PAAM/chitosan hydrogel was applied to the absorption of As(V) due to its free hydroxyl (-OH) and amino groups (-NH_2_, -NH_3_^+^), with potential adsorption properties of arsenates.

**Figure 9:**
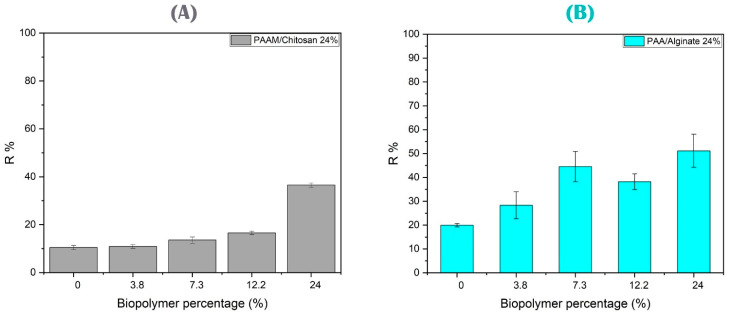
(**A**) Study of different biopolymer percentages of PAAM/chitosan gels using 0.05 g of 10 mg/L As(V) solution at pH 4, with a 90 min contact time. (**B**) Study of different biopolymer percentages of PAA/alginate gels using 0.05 g of 10 mg/L Cu(II) solution at pH 4, with a 90 min contact time.

In this study, PAAM/chitosan gels were applied with different percentages of chitosan (0% to 24 wt.%) and their absorption of arsenate was studied [71,72]. In Figure 9A, we can see that increasing the chitosan percentage increased the adsorption capacity of As(V), and we note that the bio-adsorbent material with 24% wt. chitosan was superior to adsorbents of acrylamide without chitosan [3]. Therefore, each relative increase in chitosan increased the absorption of arsenate anions. The effective use of chitosan-based compounds to remove arsenic is due to the formation of a positively charged surface that can absorb negatively charged arsenic species through both electrostatic attraction and surface complexing. Chelation is the most common binding mechanism by chitosan, either as a ‘bridge model’ or a ‘pendant model’, to describe adsorption. Nitrogen atoms in chitosan amine groups can create covalent bonds with arsenic ions at pH < 6.5 [73,74,75]. Chitosan can be effective in the adsorption of metal ions due to the presence of amino and hydroxyl groups that coordinate the electrostatic interaction sites for adsorption of As(V), including amino, hydroxyl, and amide groups involved in the removal process. Arsenic is present as oxyanions (H_2_AsO_4_^−^, HAsO_4_^2−^, AsO_4_^3−^) in aqueous media and is adsorbed on the amino and proton hydroxyl groups on the surface of chitosan [19,76].

Figure 9B shows the removal capacity of copper ions by PAA/alginate hydrogels. The results showed that a higher alginate percentage reflected a higher adsorption capacity of Cu(II). Alginate is a biopolymer composed of α-l-guluronate and β-d-mannuronate. It is a polysaccharide and mainly contains three different functional groups: -COOH (carboxylate), -C-O-C- (ether), and -OH (hydroxyl) [3,77,78]. The removal of copper ions can be explained by the higher percentage of alginates in the polyacrylic acid matrix, which increased the total chelating agents to copper ions. Cu(II) was removed from the solutions using a PAA hydrogel with an optimum percentage of 24% alginate biopolymer [79]. On the other hand, Cu(II) contains a large number of empty orbitals and is ready to receive an electron-rich group to complete the chelation process. The carboxyl and carbonyl groups inside the polymeric networks can be used as ligands to capture these heavy metals by complexation. During adsorption, Cu(II) and chelation coexist, as shown in Equations (3) and (4) [80,81]:

RCOOH ↔ RCOO^−^ + H^+^ (3)

2RCOO^−^ + Cu^2+^ ↔ (RCOO)_2_Cu (4)

#### 3.4.2. Effect of pH on Arsenic and Copper Ion Removal

**Figure 10:**
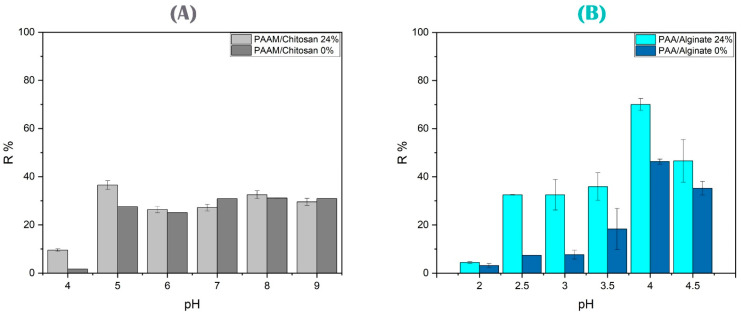
Removal of arsenate and copper ions as a function of pH solution. (**A**) Study of the pH solution effect on the adsorption of As(V) over PAAM/chitosan 24% and 0% using 0.05 g of xerogels at different pH values, from 4 to 9, and a concentration of 10 mg/L As(V) solution with a 90 min contact time. (**B**) Study of the pH solution effect on the adsorption of Cu(II) over PAA/alginate 24% and 0% using 0.05 g of xerogels at different pH values, from 2 to 4.5, and a concentration of 10 mg/L Cu(II) solution with a 90 min contact time.

To study the effect of pH on arsenate removal, the pH of the arsenate solution varied from 2 to 11 (see Figure 10A). The protonation or deprotonation on the surface of the sorbent material will also depend on the pH of the solution. The role of pH is of great importance, especially in those systems where both the absorbent and the pollutant are ionic in nature and their ionic charge is highly dependent on the pH [82]. This has a significant effect on the removal profiles of arsenic species using PAAM and PAAM/chitosan gels. Above pH 5, the arsenate removal capacity increased until 30% and 40%, mainly due to the positive charge of PAAM/chitosan 24% and the negative charge of arsenate. As(V) adsorption may occur through the surface charge of the adsorbent materials, and the anion binding gradually changes by changing the pH, causing electrostatic repulsion [83,84,85,86]. When the pH was 5, it improved the electrostatic interaction between the adsorbent ions and arsenic (HAsO_4_^2−^, H_2_AsO_4_^−^) because NH_2_ was protonated. At pH values higher than 5, the arsenate removal capacity slightly decreased and remained constant. The arsenic adsorption slightly decreased at basic pH values (pH > 10) because the surface charge of the solid was mainly negative, so repulsion occurred with the arsenate negative charge. As(V) is present in different ionic species according to the pH [9,86] (see Equations (5)–(7)).

H_3_AsO_4_ ↔H^+^ + H_2_AsO_4_^−^; pK1 = 2.3 (5)

H_2_AsO_4_^−^ ↔ H^+^ + HAsO_4_^2−^; pK2 = 6.8 (6)

HAsO_4_^2−^ ↔ H^+^ + AsO_4_^3−^; pK3 = 11.6 (7)

Arsenic exists as H_3_AsO_4_ in very acidic media, while at pH 2.3, it began to dissociate and formed negatively charged H_2_AsO_4_^−^ ions, and the H_2_AsO_4_^−^ species dissociated into HAsO_4_^2−^ at neutral pH values. Above pH 9, HAsO_4_^2−^ with OH^−^ was the main anionic species and tended to reduce arsenic uptake due to electrostatic repulsion between the negatively charged surface and anionic arsenic [9].

To study the effect of pH on Cu(II) removal (see Figure 10B), the hydrogels of alginate and acrylic acid polymers were placed in a medium with different pH values (from pH 2 to pH 5). At pH values higher than 5, the hydrolysis of copper cations and the formation of copper precipitates of (Cu(OH)_2_) occur [87]. It is necessary to study the effect of pH on the removal of Cu(II) because it affects the surface charge of the adsorbent and the charge of the metals, whereby the difference in absorption is due to the difference in pH, mainly by electrostatic repulsion between the metal cations and the gels, where charge repulsion occurs. At a low pH, the functional groups of the polymers are protonated, and the pH difference affects the high electron density on the dissolved molecules. The absorption is higher at pH 4, and the absorption decreases at lower and higher pH levels [88,89]. This is because the competitiveness of H^+^ with Cu(II) in aqueous solution and their repulsion with Cu(II) during the adsorption process will lead to strong electrostatic repulsion of heavy metals through the release of hydrogen and the formation of carboxylate anions that bind copper ions at pH 4. At this point, the dissociation of the negatively charged functional groups occurs through the dissociation of H^+^ of the carboxyl group (-COOH) and the hydroxyl group (-OH), and the carboxyl groups have a pKa of approximately 3.65 [90,91]. This means that the carboxyl groups are deprotonated and Cu(II) species are predominantly cationic, which leads to the force of attraction between the surface of the gels and the cationic copper to increase the adsorption. In addition, the color of the gels turns blue after Cu(II) adsorption, as it has the most adsorption sites and surface electrostatic effects, resulting in an increase in the adsorption capacity [92,93].

#### 3.4.3. Effect of the Amount of Adsorbent on Arsenic and Copper Ion Removal

**Figure 11:**
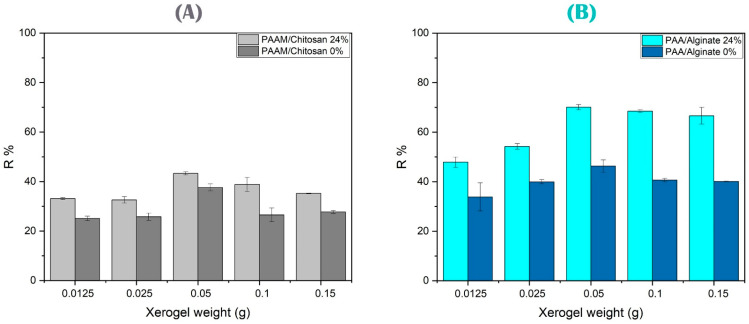
(**A**) Study of different xerogel weights of PAAM/chitosan 24% and 0% gels using 0.05 g of 10 mg/L As(V) solution at pH 5 with a 90 min contact time. (**B**) Study of different xerogel weights of PAA/alginate 24% and 0% gels using 0.05 g of 10 mg/L Cu(II) solution at pH 4 with a 90 min contact time.

Different amounts of xerogels were studied to obtain better absorption. In Figure 11A, we note that an increase in grams of xerogel used led to a change in the absorption of As(V). The nitrogen content remained constant, and the increase in the number of active sites affected the surface of the absorbent material, which had a profound effect on the adsorption behavior due to the porous structure of the smooth microparticles with a rough surface on the gels, thus changing the adsorption capacity [94,95,96,97].

The same study of the gram quantity of xerogels was performed for PAAM/alginate gels (see Figure 11B) to remove Cu(II) ions. Copper ions were independently adsorbed to the surface of the polymers due to the increase of hydroxide (-OH) and carboxylic (-COOH) groups from alginate, and this is why the increase of the adsorption increased with the surface area and with the increase of the initial amounts of grams of bio-absorbents. The number of available active sites increased, and thus, the absorption of Cu(II) ions increased to obtain a higher value due to the increased driving force of the concentration gradient of the adsorption process, which led to the maximum possible removal of metal ions [98,99].

#### 3.4.4. Effect of Contact Time on Arsenic and Copper Removal

**Figure 12:**
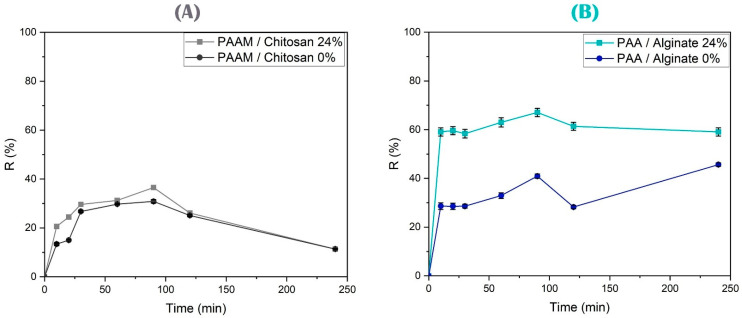
(**A**) Study of the effect of the contact time on the adsorption of As(V) over PAAM/chitosan 24% and 0% using 0.05 g of xerogels at pH 5 and a concentration of 10 mg/L As(V) solution for different contact times. (**B**) Study of the effect of the contact time on the adsorption of Cu(II) over PAA/alginate 24% and 0% using 0.05 g of xerogels at pH 4 and a concentration of 10 mg/L Cu(II) solution for different contact times.

The effect of the contact time between PAAM/chitosan and As(V) ions was tested from 10 min to 4 h (see Figure 12A). The removal capacity of As(V) increased up to 90 min. Then, the adsorption of As(V) can be explained by the fact that with the increase in contact time, diffusion can be increased; that is, arsenic ions move from the site where they are to the interior of the hydrogel [100]. After 90 min of contact time, the equilibrium state was reached due to the saturation of the active sites of the absorbent. During adsorption, there is rapid ion-exchange followed by chemical absorption, which causes a decrease in the arsenic concentration in water [101]. The rapid adsorption kinetics of nitrogen and hydroxyl groups, high surface area, low resistance, and adsorption reactions between As(V) and polymer surfaces are generally the most important kinetic controls [102,103].

To study the contact time, which ranged between 10 min and 4 h (see Figure 12B), the equilibrium adsorption capacity of alginate and acrylic acid gels for Cu(II) was explored. The adsorption of divalent metals, such Cu(II), on alginate as a component of the sorbent usually occurs as a result of ion-exchange with the participation of carboxyl groups that have different electrostatic interactions with Cu(II) ions, where a bidentate complex is formed [104,105]. The results showed that the adsorption behavior and equilibrium were achieved at approximately 90 min, which was considered the most appropriate contact time, and no significant improvement in the adsorption rate was observed after this time [106]. This is attributed to the initial abundance of unoccupied adsorption sites on the surface of the adsorbents. After the equilibrium period, the amount of Cu(II) adsorbed did not significantly change with the passage of time. The removal efficiency rapidly increased in the initial stage due to the availability of active binding sites on the sorbent material [107,108]. The adsorption occurred quickly, and equilibrium was achieved within 10–20 min. The semi-IPN is a crosslinked polymer that has the tridimensional shape of an interconnected network. Alginate has plenty of -COOH groups, which can be considered as adsorption sites for ion-exchange and chelation of Cu(II) ions to form a neutral species and establish equilibrium. The difference in the ionic sizes of the metals and the nature and distribution of the active groups need time for the equilibrium process [109].

#### 3.4.5. Effect of the Initial Arsenic or Copper Ion Concentration on Their Removal

**Figure 13:**
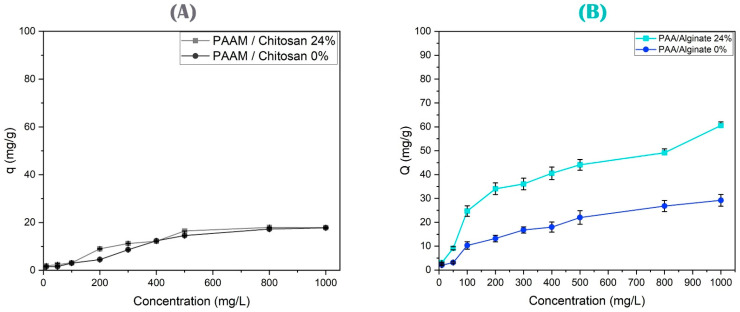
(**A**) Study of high concentrations of As(V) over PAAM/chitosan 24% and 0% using 0.05 g of xerogels at pH 5, with a 90 min contact time and different concentrations of As(V) solution. (**B**) Study of high concentrations of Cu(II) over PAA/alginate 24% and 0% using 0.05 g of xerogels at pH 4, with a 90 min contact time and different concentrations of Cu(II).

The effect of high concentrations of As(V) (between 10 and 1000 mg/L) was studied on PAAM/chitosan gels under the optimum conditions to determine the maximum absorption capacity (see Figure 13A). The maximum adsorption capacity of PAAM/chitosan gels was examined for As(V), and we noted the gradual increase in the absorption capacity with the increase in the concentration of ions; then, the absorption capacity stabilized. Functionality increased with the increasing concentration up to 1000 mg/L, and reached the final saturation stage, and the absorption was then evaluated, assuming that As(V) adsorption should be attributed to the surface complexing mechanism [110,111].

An example of arsenic removal at higher concentrations is when particles were prepared from low-cost, positively charged clay materials, consisting of Mg/Al-layered double hydroxide (Mg/Al-LDH) intercalated with MoS_4_^2−^ (MoS4-LDH), and used to remove arsenic at concentrations (10–400 mg/L) at pH > 6. They showed a capacity of 56 mg/g and were estimated to show >93% removal within 1 min and >96% removal within 5 min and 24 h. The contact time achieved 99.53% removal in 48 h [112]. Another example of arsenic removal is through the use of chitin, chitosan, and chitosan crosslinked to benzoquinone by using a chitosan concentration of 1 g/L. The removal of arsenic at pH 4 was from the initial concentrations of 400 mg/L, the removal capacity was 58 mg/g, and the removal efficiency was 30% [113]. Another example of arsenic removal using Ligand 3-impregnated Amberlite XAD-4 resin, at an arsenic concentration of 250 mg/L and at a pH of 4, obtained results with an absorption capacity of 289 mg/g and a removal efficiency of 99% in liquid-liquid and solid-liquid extraction studies [114]. 

In another study, when a metal frame was used, the best removal results were obtained. The mineral-organic frame was water-stable zirconium (UiO-66), which was used to remove arsenic at a pH of 2 and a concentration of 500 mg/L, and the removal capacity was 280 mg/g higher by the Zr6 group by forming Zr-O-As coordination bonds. Another example involves the removal of arsenic on metallic materials, an absorbent material consisting of Ni-Al-Fe. When the pH was adjusted to 7.0, the adsorption capacity was 103 mg/g, and the removal efficiency was 90.0% at low concentrations of 100 mg/L and less (0.1 mg/L) [115].

Concentrations of Cu(II) between 10 and 1000 mg/L were also studied on PAA/alginate gels under the optimum conditions to determine the maximum adsorption capacity (see Figure 13B). The results can be explained by the fact that the higher concentration of the solution makes the movement of ions easier, and then the absorption capacity tends to stabilize or decrease [116,117]. This is because most of the adsorption sites were not occupied in the initial stage, and the adsorption sites remained unsaturated during the adsorption reaction, but the saturation increased as the copper was absorbed. Three types of coordination complexes between carboxyl groups (-COO^−^) and metal ions occurred: unknown complexes, dichotomous bridging complexes, in which each of the oxygens of the -COO^−^ group were coordinated with the metal in a chelated structure, and bridge-type complexes, where the two oxygens of the COO^−^ group were coordinated with different ions after the optimum dose was reached [118,119,120,121]. The adsorption capacity remained constant due to the crowding of the adsorbent molecules, and larger concentrations cannot be absorbed because of the difficulty of forming complex ions, or a bridge of ions, with COOH and -OH groups. In addition, the protons compete with metal ions for carboxyl groups. The carboxyl groups are more complexing with Cu(II) compared to the rest of the metal ions [122,123,124]. Table 2 summarizes the maximum sorption capacities reported for sorbents to remove As(V) and Cu(II) from water.

#### 3.4.6. Regeneration and Elution Study

**Figure 14:**
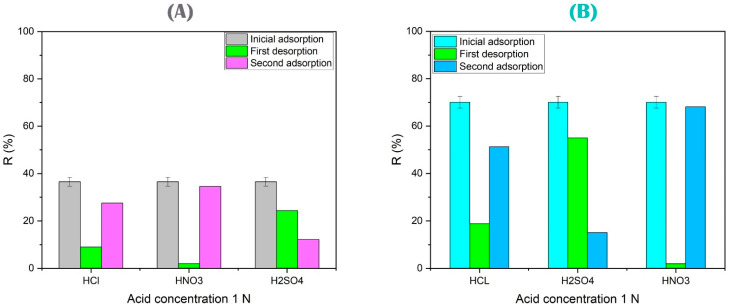
(**A**) Elution for PAAM/chitosan 24% using different acids. (**B**) Elution for PAA/alginate 24% using different acids.

**Figure 15:**
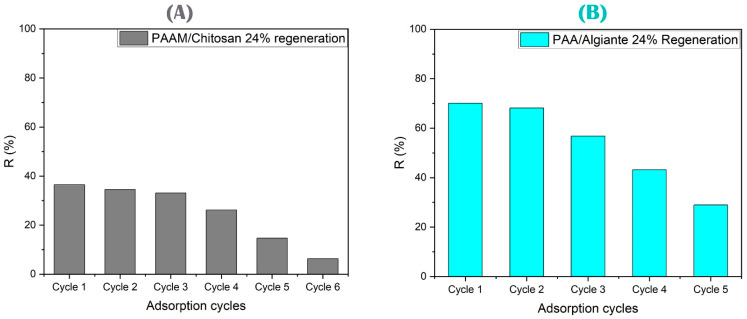
(**A**) Regeneration cycles for PAAM/chitosan 24% using different acids. (**B**) Regeneration cycles for PAA/alginate 24% using different acids.

Elution is the study of the elute solution and determining the best solution that is placed to restore the effectiveness of the gel, and the As(V) and Cu(II) ions absorbed on it are removed by acids (see Figure 14). All the metal ions were desorbed using acids such as HCl, HNO_3_, and H_2_SO_4_. However, the regeneration results remained similar, and the adsorption capacity was maintained in six cycles for PAAM/chitosan 24%. The decrease was evident at the fourth cycle, where the adsorption capacity after four regeneration cycles remained almost constant and where nitric acid probably destroyed the active adsorbent group (see Figure 15) [144,145].

R-NH_3_^+^ H_2_AsO_4_^−^ + HNO_3_ ↔ R-NH_3_^+^ NO_3_^−^ + H_3_AsO_4_ (8)

R-NH_3_^+^ H_2_AsO_4_^−^ + H_2_SO_4_ ↔ R-NH_3_^+^ SO_4_^−2^ + H_3_AsO_4_ (9)

R-NH_3_^+^ H_2_AsO_4_^−^ + HCl ↔ R-NH_3_^+^ Cl^−^ + H_3_AsO_4_ (10)

Nitric acid was found to be an effective regenerator for As(V) and Cu(II). Nitric acid contains a nitrogen atom. This element helps in the formation of Cu(NO_3_)_2_ and complexes with arsenic that can help in the reuse of gels. Nitric acid is better than sulfuric acid and hydrochloric acid. This is because the NO_3_ group has a better desorption ability to desorb many types of heavy metals. On the other hand, sulfuric acid dissociates to SO_2_ and does not form CuSO_4_. Hydrochloric acid forms copper complexes and does not form CuCl_2_ (see Equations (11)–(13)) [146].

Both gels are weak bases and are promising absorbent materials for As(V), where the weak-base Lewis sulfate was coordinated with As(V) and then the regeneration process was performed. On the other hand, the adsorption–desorption of PAA/alginate 24% with Cu(II) from an aqueous solution of alginate containing the carboxyl anionic group had a maximum recovery percentage of approximately 97% (see Figure 15) when 1 mol/L of HNO_3_ solution was used [147,148].

RCOO–Cu^2+^ + HNO_3_ ↔ RCOO– H^+^ + Cu(NO_3_)_2_ (11)

RCOO–Cu^2+^ + H_2_SO_4_ ↔ RCOO– H^+^ + CuSO_4_ (12)

RCOO–Cu^2+^ + HCl ↔ RCOO– H^+^ + CuCl_2_ (13)

## 4. Conclusions

The innovation of this project is that with a higher mg/g for 10 mL of solution and the ability to be reused by HNO_3_, the manufacture of hydrogels was successfully achieved using PAAM with chitosan and PAA with alginate with a semi-IPN structure. The study of the removal of As(V) ions by xerogels from PAAM/chitosan showed that the best amount of chitosan biopolymer was 24% and the best pH value was 5.0 for arsenic adsorption. The best agitation contact time was 90 min, 50 mg of xerogel was sufficient, and when a high arsenic concentration of 1000 mg/L was applied, the hydrogel was found to have an adsorption capacity of 20 mg/g. It could be reused up to six times, and the best acid for eluting and reusing was nitric acid. When studying the adsorption of PAA/alginate, it was found that the best ratio of the polymer to the alginate was 24% and that it had the best absorption of copper at a pH of 4.0. Here, 50 mg of weight was sufficient to absorb an initial concentration of Cu(II) and the best contact time with the copper solution was 90 min. At a copper concentration of 1000 mg/L, the results showed that the hydrogel had an adsorption capacity of 67 mg/g. The hydrogel had the ability to be reused five times, but by the fourth time it maintained its concentration. The best acid for eluting and reuse was nitric acid.

## Figures and Tables

**Table 1:** Details of the amounts of reagents used in each synthesis and their yields (%).

**Sample No.**	**Percentage ** **(%)**	**PAA (mL)** **± 0.005 mL**	**MBA (g) ± 0.005 g**	**PSA (g) ± 0.0005 g**	**Alginate (g) ± 0.005 g**	**Yields (%)**
1	0	1.37 (1.489 g)	0.3114	0.0116	0	94.3
2	3.8	1.37 (1.489 g)	0.3114	0.0116	0.07107	96.63
3	7.3	1.37 (1.489 g)	0.3114	0.0116	0.14214	97.12
4	12.3	1.37 (1.489 g)	0.3114	0.0116	0.28428	98.6
5	24	1.37 (1.489 g)	0.3114	0.0116	0.56856	94.46
6	40	1.37 (1.489 g)	0.3114	0.0116	1.208	91.33
7	55.66	1.37 (1.489 g)	0.3114	0.0116	2.27424	95.46
**Sample No.**	**Percentage ** **(%)**	**PAM (g) ± 0.005 g**	**MBA (g) ± 0.005 g**	**PSA (g) ± 0.0005 g**	**Chitosan (g) ± 0.005 g**	**Yields (%)**
8	0	1.4412	0.3114	0.0116	0	94.6
9	3.9	1.4412	0.3114	0.0116	0.0720	95.7
10	7.6	1.4412	0.3114	0.0116	0.14412	99.1
11	14	1.4412	0.3114	0.0116	0.28824	99.75
12	24	1.4412	0.3114	0.0116	0.57648	96.02
13	40	1.4412	0.3114	0.0116	1.1761	96.4
14	60	1.4412	0.3114	0.0116	2.6463	94.7

**Table 2:** Maximum adsorption capacity of reported adsorbents for Cu(II) removal from water.

**Adsorbent**	**Adsorbate**	**Adsorption Capacity (mg/g)**	**pH**	**Concentration** **(mg/L)**	**R%**	**Ref.**
3D network nanostructured sodium alginate	Cu(II)	13.38	7	1000	82.73	[122]
Alginate/polyethyleneimine	Cu(II)	322.6	5.5	200	45.7	[125]
Chitosan/sodium alginate/calcium ion	Cu(II)	70.83	3	20–100		[126]
Sodium alginate-grafted polyacrylamide/graphene oxide	Cu(II)	68.76	5	25–250	80	[127]
Alginate-modified graphitic carbon nitride	Cu(II)	168.2	6	500	95	[128]
Alginate/starch ether composite	Cu(II)	247.16	4	100–1000	78.03	[129]
Carbon nanotube/calcium alginate composites	Cu(II)	25.81	5.5	25–250	92.21	[130]
Calcium alginate-immobilized kaolin	Cu(II)	47.46	5	15	79.43	[131]
Mxene/alginate	Cu(II)	87.6	5	10	63.5	[132]
Sodium alginate supported on melamine sponge	Cu(II)	83.0		50–250	66.6	[133]
Alginate-immobilized bentonite	Cu(II)	114.70	4	400		[134]
Alginate-based porous nanocomposite hydrogels	Cu(II)	87.2	3	20–500		[135]
Strength-toughness alginate composite fibers	Cu(II)	102.4		10	78.6	[136]
alginate-polyethyleneimine hybrid aerogel	Cu(II)	214.4	4	--	95.1	[137]
PAA/alginate 24%	Cu(II)	63.59	4	1000		This study
**Adsorbent**	**Adsorbate**	**Adsorption Capacity (mg/g)**	**pH**	**Concentration (mg/L)**	**R%**	**Ref.**
Chitosan thiomer	As(V)	17.66	6–8	0.050	87%	[138]
Chitosan and nanochitosan	As(V)	39.68	4	0.250–11		[139]
Chitosan-related electrospun nanofiber	As(V)	0.5	7.2	0.1–0.75	3%	[140]
Iron-functionalized chitosan-based electrospun nanofiber	As(V)	11.2	7.2	0.1–0.75	98%	[140]
Chitosan-based MCS/ZnO@Alg gel microspheres	As(V)	63.69	4	10	99%	[141]
Core–shell/bead-like ethylenediamine-functionalized Al-pillared montmorillonite/calcium alginate	As(V)	61.94	4	50	94.85%	[142]
Magnetic nanoparticle-impregnated chitosan beads	As(V)	35.7	6.8	2000	88.2%	[143]
PAAM/chitosan 24%	As(V)	17.8	5	1000		This study

## Data Availability

All the data are available within the manuscript.
